# River Surface Velocity Measurement for Rapid Levee Breach Emergency Response Based on DFP-P-LK Algorithm

**DOI:** 10.3390/s24165249

**Published:** 2024-08-14

**Authors:** Zhao-Dong Xu, Zhi-Wei Zhang, Ying-Qing Guo, Yan Zhang, Yang Zhan

**Affiliations:** 1 China-Pakistan Belt and Road Joint Laboratory on Smart Disaster Prevention of Major Infrastructures, Southeast University, Nanjing 210096, China; 2 College of Mechanical and Electronic Engineering, Nanjing Forestry University, Nanjing 210037, China

**Keywords:** river surface velocity, feature point fusion detection, dynamic update mechanism, optical flow estimation

## Abstract

In recent years, the increasing frequency of climate change and extreme weather events has significantly elevated the risk of levee breaches, potentially triggering large-scale floods that threaten surrounding environments and public safety. Rapid and accurate measurement of river surface velocities is crucial for developing effective emergency response plans. Video image velocimetry has emerged as a powerful new approach due to its non-invasive nature, ease of operation, and low cost. This paper introduces the Dynamic Feature Point Pyramid Lucas–Kanade (DFP-P-LK) optical flow algorithm, which employs a feature point dynamic update fusion strategy. The algorithm ensures accurate feature point extraction and reliable tracking through feature point fusion detection and dynamic update mechanisms, enhancing the robustness of optical flow estimation. Based on the DFP-P-LK, we propose a river surface velocity measurement model for rapid levee breach emergency response. This model converts acquired optical flow motion to actual flow velocities using an optical flow-velocity conversion model, providing critical data support for levee breach emergency response. Experimental results show that the method achieves an average measurement error below 15% within the velocity range of 0.43 m/s to 2.06 m/s, demonstrating high practical value and reliability.

## 1. Introduction

In recent years, global climate change has intensified, and extreme weather events have become frequent. According to the latest report of the United Nations Intergovernmental Panel on Climate Change (IPCC), the frequency and intensity of extreme rainfall events have increased significantly over the past 50 years. This trend has led to a significant increase in flood risk, particularly the growing threat of levee breaches. Embankment breaches not only cause large-scale flooding, posing a serious threat to the surrounding environment and people’s lives and property, but also cause significant damage to infrastructure and ecosystems. In this context, rapid and accurate measurements of river surface flow velocity are essential for assessing the risk of embankment breaches and for developing and implementing effective rescue measures [1,2,3].

Traditional methods of river flow measurement (e.g., buoy method, velocimetry pole method, and stream gauge method) face many challenges and limitations in practical applications. The applicability of these methods is significantly limited in the case of complex river segments or large-scale monitoring, which may interfere with the river environment and make it difficult to cover large areas efficiently [4,5]. In addition, with traditional methods it is usually difficult to provide continuous monitoring data over a long period of time and at a high frequency, which affects the timely mastery of river dynamic changes. These limitations are particularly prominent in the face of emergencies such as dike-breach rescue, mainly in terms of operational safety and response speed: under hazardous conditions such as floods, these methods increase the risk of direct contact between staff and turbulent water, which poses a major safety hazard. At the same time, they are often difficult to deploy rapidly and cannot obtain much-needed real-time data in a timely manner, which seriously restricts the efficiency and safety of flood prevention and mitigation.

Faced with these challenges, researchers have begun to seek safer and more efficient alternatives. With the rapid development of technology, non-contact measurement methods have gradually become a research hotspot. These methods, which do not require direct contact with the water body, not only dramatically reduce the risk of personnel safety, but also reduce the wear and tear of equipment and maintenance costs, bringing new possibilities for river flow measurement. Among the many non-contact methods, the study by Costa et al. [6] demonstrated the feasibility of converting surface velocity data to mean velocity, laying the foundation for the development of this field. In 2008, Mandlburger et al. [7] further optimized a LiDAR-based terrain model, demonstrating its vast potential for application in flow-velocity and flow-rate measurements. However, these methods still face some challenges such as high equipment cost and unstable measurement accuracy in specific environments. For example, the accuracy of radar data may be affected in strongly fluctuating and turbulent environments [8], and the accuracy of LiDAR measurements can be significantly degraded in the presence of strong light reflections.

Video image velocimetry techniques have received widespread attention due to the advantages of simple equipment, lower cost, and wide monitoring range [9]. These methods capture the motion of water body surface features (e.g., floating objects, surface texture, etc.) through camera equipment and utilize advanced image processing techniques (e.g., optical flow method, feature point tracking, etc.) to calculate the flow velocity [10]. Common video image velocimetry methods include Particle Image Velocimetry (PIV), Large Scale Particle Image Velocimetry (LSPIV) [11], the LK optical flow method, and feature point tracking. In recent years, researchers have explored and innovated in this field in various ways. Zhao et al. [12] proposed a flow-velocity measurement method based on inter-frame differencing and template matching, which performs well in stable environments but is susceptible to interference in dynamic environments and weakly adapted to changes in illumination. Feng Quan et al. [13] utilized high-definition cameras and machine vision technology to achieve non-contact, high-precision flow-rate measurements, which has the advantages of real-time and wide applicability but is still sensitive to changes in ambient light and suffers from the problem that feature points are easily lost. The video-image flow-rate estimation technique proposed by Bradley et al. [14] demonstrates adaptability in different water flow environments, but it has the advantage of high adaptability in complex lighting conditions and dense floating objects, and its accuracy still faces challenges. Detert et al. [15] proposed a low-cost image-based method for weather airborne river flow measurements, which simplifies the equipment requirements and operational procedures, enabling effective river flow measurements even in small- and medium-sized research projects. Fujita et al. [16] applied the LSPIV technique to measure flow velocity and flow rate during a flood event by using an unmanned aerial vehicle (UAV) with a high-resolution video camera, demonstrating its potential in emergency monitoring. Although each of these methods has its own characteristics and demonstrates advantages in different application scenarios, they still face some common challenges, such as the sensitivity to environmental conditions and the accuracy problem in complex water flow situations, which points out the direction for future research.

To summarize, video image velocimetry methods have the advantages of being non-intrusive, easy to operate, used in real-time, and low cost; however, how to improve the robustness and accuracy of video image velocimetry and ensure the reliability of the algorithms in complex environments are still the focus of the current research and difficult problems. In this paper, a dynamic feature point updating strategy based on the pyramid hierarchical LK optical flow method (DFP-P-LK) speed measurement method is proposed. The method integrates the pyramid hierarchical structure, dynamic feature point updating strategy, and feature point fusion detection mechanism, aiming to improve the stability and accuracy of the optical flow method in complex scenes, and optimize the robustness and accuracy of the optical flow estimation. The performance of the DFP-P-LK algorithm is first evaluated using a public dataset. Then, it is validated on a homemade water flow dataset by combining the optical flow-velocity conversion model, image preprocessing, and camera calibration techniques. The test results demonstrate the applicability and reliability of the method in different complex scenarios, showing that it outperforms the traditional method in all kinds of tests, proving its potential in practical applications and providing an effective reference for further improvement of the optical flow method.

## 2. Essential Basic Knowledge

### 2.1. Optical Flow-Velocity Conversion

To measure surface flow velocity using the optical flow method, it is necessary to construct an optical flow-velocity conversion model that transforms the estimated optical flow motion on the river surface into actual motion velocity. First, camera calibration is utilized to establish a mapping relationship between a point $P(P_{x},P_{y})$ in the two-dimensional image and a point W(X,Y,Z) in the three-dimensional world coordinate system:

(1) $$\left[\begin{array}{c} P_{x}\\ P_{y}\\ 1 \end{array}\right]=\left[\begin{array}{ccc} \frac{f}{dx} & 0 & P_{x0}\\ 0 & \frac{f}{dy} & P_{y0}\\ 0 & 0 & 1 \end{array}\right]\left[\begin{array}{c} X\\ Y\\ Z \end{array}\right]=\left[\begin{array}{ccc} f_{x} & 0 & P_{x0}\\ 0 & f_{y} & P_{y0}\\ 0 & 0 & 1 \end{array}\right]\left[\begin{array}{c} X\\ Y\\ Z \end{array}\right]=K\left[\begin{array}{c} \frac{X}{Z}\\ \frac{Y}{Z}\\ 1 \end{array}\right]$$

where f is the camera’s focal length in millimeters; dx and dy denote the length and width of a single pixel in millimeters, respectively; $\left(P_{x0},P_{y0}\right)$ is the coordinate of the camera’s optical axis center on the imaging plane; and K is the camera’s intrinsic parameter matrix, which can be solved using Zhang’s calibration method [17].

Consider a point W on the water surface that moves from $W_{1}(X_{1},Y_{1},Z)$ to $W_{2}(X_{2},Y_{2},Z)$ over a time interval ∆t. Its corresponding point in the image plane moves from $P_{1}(P_{x1},P_{y1})$ to $P_{2}(P_{x2},P_{y2})$.

Assuming that the surface of the river at the time of shooting is perpendicular to the camera imaging plane, it can be assumed that $Z=Z_{1}=Z_{2}$ holds for a short period of time, where Z is the distance of the horizontal surface from the camera lens. Then, the relationship between the image optical flow $\left({∆P}_{x},{∆P}_{y}\right)$ and the actual optical flow (∆X,∆Y) of this point can be expressed as:

(2) $$\left\{\begin{array}{c} {∆P}_{x}=P_{x2}-P_{x1}=\frac{f_{x}}{Z}\left(X_{2}-X_{1}\right)=\frac{f_{x}}{Z}∆X\\ {∆P}_{y}=P_{y2}-P_{y1}=\frac{f_{y}}{Z}\left(Y_{2}-Y_{1}\right)=\frac{f_{y}}{Z}∆Y \end{array}\right.$$

Thus, an optical flow-velocity conversion model can be constructed, where the actual motion velocity of this point is given by:

(3) $$v_{0}=\frac{∆W}{∆t}=\frac{\sqrt{{\left(X_{2}-X_{1}\right)}^{2}+{\left(Y_{2}-Y_{1}\right)}^{2}+{\left(Z_{2}-Z_{1}\right)}^{2}}}{∆t}=\frac{Z}{∆t}\sqrt{\frac{1}{f^{2}}{{∆P}_{x}}^{2}+\frac{1}{f_{y}^{2}}{{∆P}_{y}}^{2}}$$

where ∆W is the actual distance traveled by point W over the time interval ∆t, $f=\frac{1}{2}(f_{x}+f_{y})$.

### 2.2. LK Optical Flow Method

The fundamental principle of the optical flow method is that the gray value of the corresponding pixel point remains constant between neighboring images [18]. Specifically, suppose that at moment t, the gray value of the image position (x,y) is I(x,y), and the optical flow at this point is (u,v), which denotes the speed of movement of the point in the x and y directions. After a very short time interval δt, at the moment t+δt the point moves to (x+δx,y+δy), which is obtained according to the assumption of grayscale invariance:

(4) $$I\left(x,y,t\right)=I(x+\delta x,y+\delta y,t+\delta t)$$

Assuming a small displacement, the Taylor expansion of Equation (4) is:

(5) $$I\left(x+\delta x,y+\delta y,t+\delta t\right)=I\left(x,y,t\right)+\frac{\partial I}{\partial x}*\delta x+\frac{\partial I}{\partial y}*\delta y+\frac{\partial I}{\partial t}*\delta t+H.O.T$$

where H.O.T represents higher-order infinitesimals.

Transforming the differential expression yields:

(6) $$\frac{\partial I}{\partial x}*\frac{\delta x}{\delta t}+\frac{\partial I}{\partial y}*\frac{\delta y}{\delta t}+\frac{\partial I}{\partial t}=0$$

Let u=δx/δt and v=δy/δt, and the basic constraint equations for optical flow extrapolation can be obtained:

(7) $$(I_{x}=\frac{\partial I}{\partial x},\;I_{y}=\frac{\partial I}{\partial y},I_{t}=\frac{\partial I}{\partial t})\;\to I_{x}u+I_{y}v+I_{t}=0$$

The LK optical flow method [19] simplifies the computation by focusing on local spatial neighborhoods. Based on the assumption of brightness constancy, it introduces additional assumptions of small displacement and neighborhood optical flow consistency to facilitate solving the basic constraint equation.

(1) Small displacement assumption: the pixel movement between adjacent frames is limited, allowing the calculation of grayscale differentials with respect to displacement based on brightness changes;
(2) Neighborhood optical flow consistency: optical flow vectors of nearby pixels maintain high consistency.

Specifically, within a very small spatial neighborhood Ω (of size n, n≥2), all pixels have the same optical flow vector and satisfy the optical flow constraint equation. Therefore, optical flow can be calculated independently for each pixel within this neighborhood. Combining the equations for all pixels in the neighborhood yields a system of m × n equations:

(8) $$\left\{\begin{array}{c} I_{x1}u+I_{y1}v+I_{t1}=0\\ I_{x2}u+I_{y2}v+I_{t2}=0\\ \vdots \\ I_{xn}u+I_{yn}v+I_{tn}=0 \end{array}\right.$$

The matrix form of Equation (8) is:

(9) $$\left[\begin{array}{cc} \begin{array}{c} I_{x1}\\ \begin{array}{c} I_{x2}\\ \vdots \\ I_{xn} \end{array} \end{array} & \begin{array}{c} I_{y1}\\ \begin{array}{c} I_{y2}\\ \vdots \\ I_{yn} \end{array} \end{array} \end{array}\right]\left[\begin{array}{c} u\\ v \end{array}\right]=-\left[\begin{array}{c} I_{t1}\\ \begin{array}{c} I_{t2}\\ \vdots \\ I_{tn} \end{array} \end{array}\right]$$

Simplifying Equation (9), where the unknown variable $\vec{x}$ represents the two components (u,v) of the optical flow vector:

(10) $$A\cdot \vec{x}=\vec{b}$$

The approximate solution to Equation (10) is:

(11) $$\vec{x}=\left[\begin{array}{c} u\\ v \end{array}\right]={\left[\begin{array}{cc} \sum_{i=1}^{n}I_{xi}^{2}\; & \sum_{i=1}^{n}I_{xi}I_{yi}\\ \sum_{i=1}^{n}I_{xi}I_{yi} & \sum_{i=1}^{n}I_{yi}^{2} \end{array}\right]}^{-1}\left[\begin{array}{c} -\sum_{i=1}^{n}I_{xi}I_{yi}\\ -\sum_{i=1}^{n}I_{yi}I_{yi} \end{array}\right]$$

In regions of the image where pixel changes occur in both directions, matrix A is invertible. However, in areas with minimal grayscale variation, x is not invertible; thus, not all optical flows can be calculated.

In optical flow tracking, since the sufficient condition for invertibility is $\left|G\right|=\left|A^{T}A\right|\neq 0$, $A^{T}A$ full rank and having two larger eigenvectors. This condition is automatically satisfied when the center of the window for optical flow tracking is a feature point, which is the reason why the LK optical flow method chooses image feature points for tracking.

### 2.3. Image Pyramid

An image pyramid is a hierarchical structure composed of images with progressively lower resolutions. This study employs a Gaussian pyramid, which reduces the scale of the original image through recursive downsampling. The bottom layer $I_{0}$ is the original image, which is downsampled to obtain images with decreasing resolution $I_{1}$, $I_{2}$, $I_{3}$, …, $I_{n}$, and generally *n* is three to five layers. The two neighboring layers satisfy a fixed scale relationship, and generally the resolution of the next layer is one-half of the upper layer.

Let L denote the number of layers of the Gaussian pyramid, e.g., the bottom image $I^{0}$, whose width and height are $n_{x}^{0}$ and $n_{y}^{0}$, respectively. The L−1 th layer image is $I^{L-1}$, and its width and height are $n_{x}^{L-1}$ and $n_{y}^{L-1}$, respectively. The formula for the Lth layer is:

(12) $$\begin{array}{cc} I^{L}\left(x,y\right)=\frac{1}{4}I^{L-1} & \left(2x,2y\right)+\frac{1}{8}\left(I^{L-1}\left(2x-1,2y\right)+I^{L-1}\left(2x+1,2y\right)\right)\\ & +\frac{1}{16}\left(I^{L-1}\left(2x-1,2y-1\right)+I^{L-1}\left(2x+1,2y+1\right)\right)\\ & +\frac{1}{16}\left(I^{L-1}\left(2x-1,2y+1\right)+I^{L-1}\left(2x+1,2y-1\right)\right) \end{array}$$

### 2.4. Pyramid-Based LK Optical Flow Method

The basic concept of the Pyramid-Based Lucas–Kanade Optical Flow algorithm (P-LK) is as follows [20]: First, an image pyramid is constructed. The optical flow estimation then begins at the top layer of the pyramid, where the lower resolution satisfies the LK assumption. The process iteratively moves downward, progressively refining the optical flow results from the upper layers. This ultimately produces an accurate optical flow field at the original image scale.

**Figure 1:**
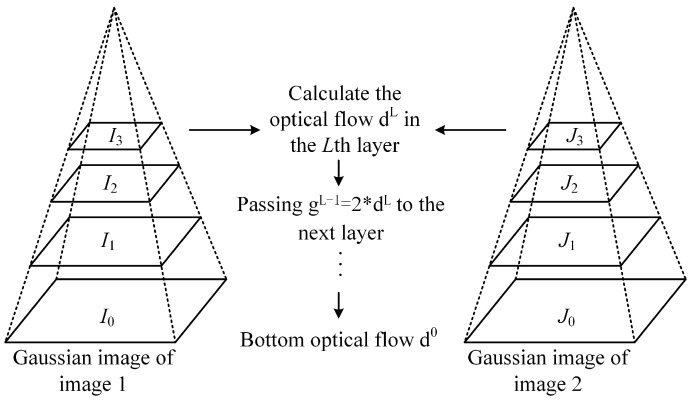
The Pyramid-Based LK optical flow method.

The Pyramid-Based LK Optical Flow method is shown in Figure 1, and the key steps are:

(1) Construction of Image Pyramids. Two image pyramids are constructed using the method described in Section 2.3;
(2) Top-Level Optical Flow Estimation. The initial optical flow is estimated using the LK algorithm at the highest level of the pyramid;
Pyramid Iteration Down Layer by Layer. The optical flow from the previous layer is propagated to the next layer, and the optical flow is scaled to match the resolution of the current layer. For example, the top-level optical flow $d^{L}$ from the previous layer to the next is $g^{L}=2*d^{L}$. Then, the optical flow estimation is refined by combining it with the image $I^{L}$ of the current layer. This process is repeated until reaching the bottom layer, which is the original image.

## 3. Dynamic Feature Point Pyramid Lucas–Kanade (DFP-P-LK) Optical Flow Method

The traditional LK optical flow method passively updates feature points, only initiating a new feature point tracking detection when all existing points have failed to track. However, due to the fluctuating and unstable nature of water flow, feature points are easily lost and unsuitable for long-term tracking. Consequently, the traditional method struggles to maintain tracking accuracy. Therefore, an active approach to updating feature points is necessary, continuously providing an ample supply of new feature points to enhance robustness in dynamic water flow environments.

### 3.1. Feature Point Fusion Detection Strategy

Feature point detection is a crucial step in computer vision, with commonly used algorithms including Harris corner detection, Scale-Invariant Feature Transform (SIFT), Speeded-Up Robust Features (SURF), and Shi–Tomasi corner detection. SURF is renowned for its efficiency and robustness, capable of detecting a large number of candidate feature points and generating descriptors with scale and rotation invariance. On the other hand, Shi–Tomasi evaluates the quality of corners by the minimum eigenvalue, making it both fast and stable. To fully leverage the advantages of both methods, this paper adopts a strategy that combines SURF and Shi–Tomasi as follows:

(1) Initially, use the SURF algorithm to obtain the set of candidate feature points from the input image I, denoted as $S=\{p_{1},p_{2},\cdots \}$;
(2) For each candidate feature point $p_{i}$, generate a descriptor that contains key information about the local region around the feature point within an L×L window, such as gradient direction and gradient magnitude;
(3) After obtaining the descriptor of the candidate feature point $p_{i}$, construct the gradient matrix according to the steps of the Shi–Tomasi algorithm, and calculate the minimum eigenvalue;
(4) After obtaining the feature descriptors for each candidate feature point $p_{i}$, construct the gradient matrix G according to the steps of the Shi–Tomasi algorithm and calculate the minimum eigenvalue $\lambda_{min}$ of G;
(5) Take the $\lambda_{min}$ as the response value of the feature point and compare the response value of the feature point with the preset threshold value. If the response value exceeds the threshold, the candidate feature point $p_{i}$ is identified as the Shi–Tomasi corner;
(6) After traversing all the candidate feature points, the final set of Shi–Tomasi corner points is obtained;
(7) According to the response values of Shi–Tomasi corner points, select the top N feature points as the final set of quality feature points.

### 3.2. Dynamic Update Strategy for Feature Points

The dynamic update of feature points consists of two aspects:

(1) Control the update and detection of feature points using the maximum consecutive processing frames $f_{max}$. When the number of consecutive processing frames $f_{num}$ in an update cycle reaches the set value $f_{max}$, force a re-detection of feature points to mitigate the volatility of surface feature points;
(2) Set the lower limit of the number of feature points $P_{min}=\alpha \times P_{max}$ to address the issue of declining feature point tracking quality and instability during long-term detection. Here, $P_{max}$ is the maximum number of detected feature points in one cycle, which is the number detected during the initial feature point detection. α is a constant between (0,1), used to control the lower limit value $P_{min}$.

**Figure 2:**
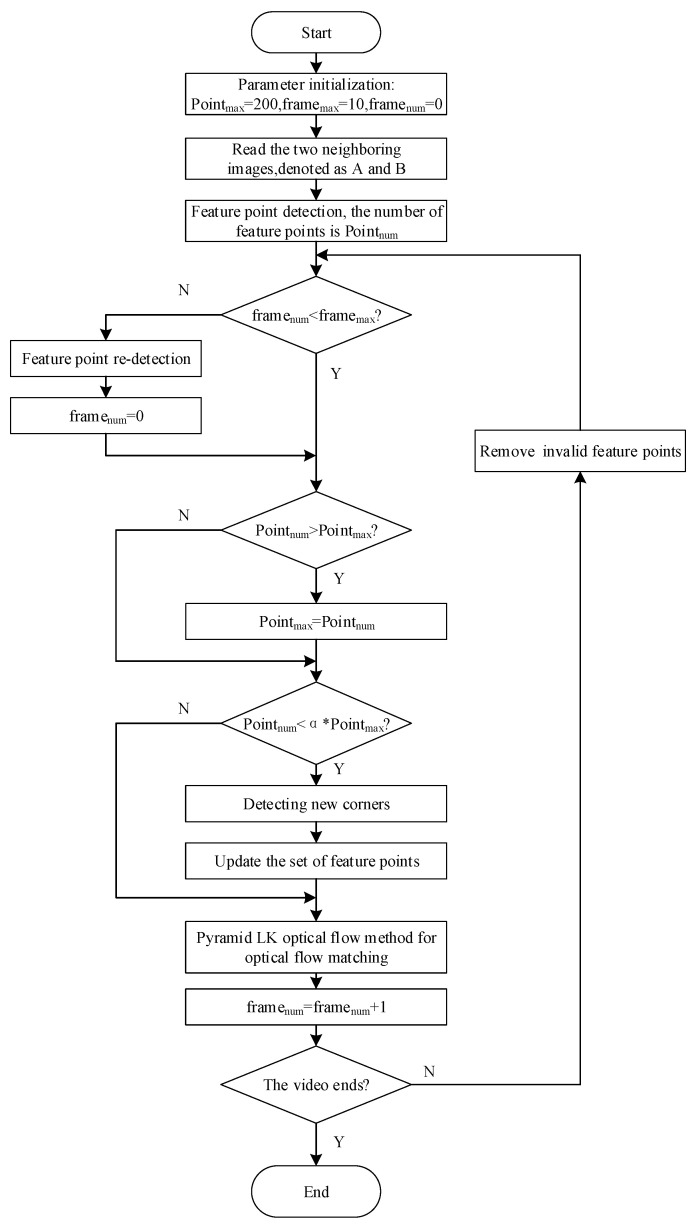
Flowchart of Dynamic Feature Point-Based Pyramid Layered Lucas–Kanade optical flow.

The flow of the improved algorithm is detailed in Figure 2.

## 4. Experimental Analysis

This experiment was completed under Windows 10, and the CPU was an Intel(R) Core(TM) i3-12100F CPU with a standard frequency of 3.30 GHz; the memory was 16 GB.

### 4.1. Detection Strategy Measurement of River Surface Flow Velocity Based on DFP-P-LK

**Figure 3:**
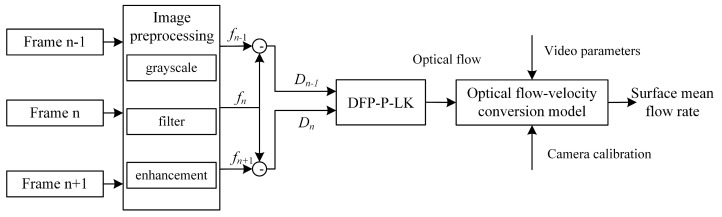
Surface flow-velocity measurement of rivers based on DFP-P-LK.

Based on the optical flow-velocity conversion model, combined with image preprocessing and the proposed DFP-P-LK optical flow estimation algorithm, the contactless measurement of river surface flow velocity can be realized. First, three consecutive frames of the video are acquired and subjected to grayscale processing, image filtering, and image enhancement to enhance the image features. Next, the preprocessed images are used to calculate the frame difference map between neighboring frames to accurately estimate the image optical flow motion of the feature points by the DFP-P-LK algorithm. Finally, combining the optical flow-velocity conversion model, camera calibration parameters, and video basic information, the image optical flow obtained by DFP-P-LK is converted into actual velocity to obtain the average flow-velocity information on the river surface. Through this series of steps, the accurate measurement of the flow velocity on the river surface can be realized to ensure the accuracy and reliability of the measurement results. The flow chart is shown in Figure 3.

### 4.2. Datasets

In this paper, the performance of the DFP-P-LK algorithm and the river surface velocimetry method based on DFP-P-LK are validated using the public dataset Flying Chairs and the homemade water flow dataset. The Flying Chairs dataset is a standard dataset for optical flow estimation, which contains a variety of synthesized image pairs and their corresponding optical flow fields, and is used to evaluate the accuracy of the optical flow algorithm.

The homemade water flow dataset comprises 82 videos captured under varying weather conditions from different sections of Sancha River and Zihu Creek in Nanjing, Jiangsu Province, China. The flow velocities range from 0.43 to 2.06 m/s, as detailed in Table 1. Videos were recorded using the DJI Mavic 2 Pro drone at a resolution of 3840 × 2160 pixels and a frame rate of 30 frames per second, with varying filming heights. Flow velocities were measured using the LS20B propeller-type current meter, with a measurement range of 0.05 to 15 m/s and an absolute relative error ≤ 5%.

### 4.3. Experimental Analysis of DFP-P-LK on Flying Chairs

#### 4.3.1. Evaluation Metrics

The mean angular error (*MAE*) and the mean endpoint error (*APE*) are used as evaluation metrics to measure the difference between the predicted optical flow and the real optical flow.

*MAE* is the average value of angular error in the range of 0 to 180 degrees, which reflects the angular deviation in direction between the predicted and real optical flow of a pixel point. When it is 0 degrees, it means that the two optical flow fields are identical, and 180 degrees means that the two optical flow fields are completely opposite.

If we define the total number of pixel points as N and the angular error $\theta_{i}$ between the predicted optical flow $u_{i}$ and the true optical flow $v_{i}$ for each pixel point i, we can then calculate the average of the angular errors of all the pixel points to obtain the *MAE*. The calculation formula is:

(13) $$\theta_{i}=\arccos(\frac{u_{i}\cdot v_{i}}{\left|u_{i}\right|\left|v_{i}\right|})$$

(14) $$MAE=\frac{1}{N}\sum_{i=1}^{N}\theta_{i}$$

*APE* is another commonly used metric for evaluating the optical flow field. It represents the average value of the endpoint error between the predicted optical flow field and the real optical flow field, and a lower *APE* value indicates a more accurate prediction of the motion amplitude.

First, the Euclidean distance between the predicted optical flow $u_{i}$ and the real optical flow $v_{i}$ is first calculated as the endpoint error $e_{i}$. Then, the average of the endpoint errors $e_{i}$ of all pixel points is calculated to obtain the *APE*. The formula is:

(15) $$e_{i}=\left\|u_{i}-v_{i}\right\|$$

(16) $$APE=\frac{1}{N}\sum_{i=1}^{N}e_{i}$$

#### 4.3.2. Test and Analysis of DFP-P-LK on Flying Chairs

**Figure 4:**
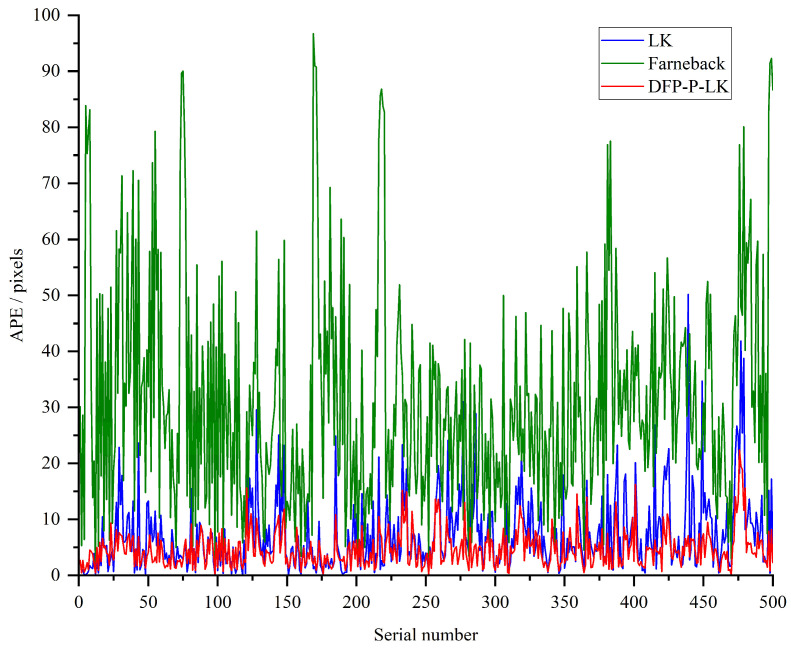
The *APE* of DFP-P-LK, LK, and Farneback methods on the Flying Chair.

**Figure 5:**
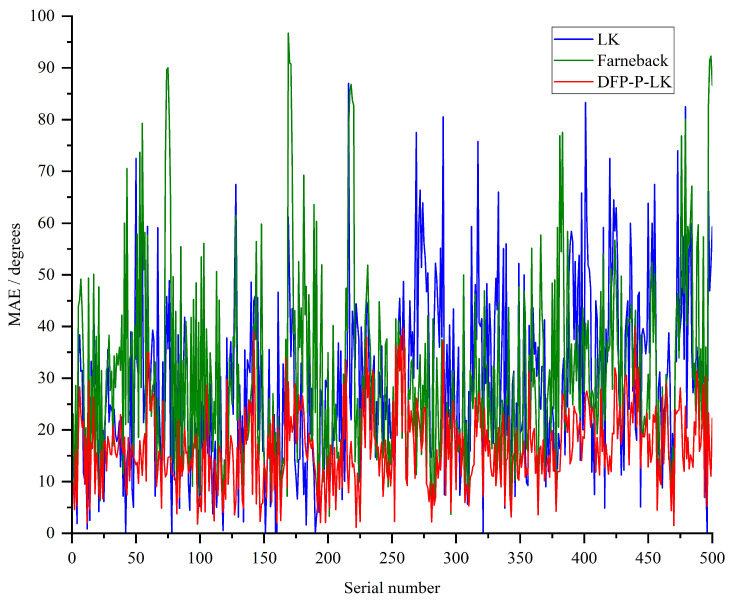
The *MAE* of DFP-P-LK, LK, and Farneback methods on the Flying Chair.

As the commonly used optical flow evaluation metrics are *MAE* and *APE*, it is expected that the values of both are as small as possible. The former is used to evaluate the angular directional deviation between the predicted and real optical flow, and smaller values indicate better directional consistency, while the latter is used for the evaluation of the endpoint error value, and smaller values indicate higher prediction accuracy. The *MAE* and *APE* results of the Dynamic Feature Point Update Strategy-Based Pyramid Hierarchical LK Optical Flow method (DFP-P-LK), LK method, and the Farneback Optical Flow method on FlyingChair are shown in Figure 4 and Figure 5, respectively.

Combining Figure 4 and Figure 5, the overall trend of *MAE* and *APE* results for the improved DFP-P-LK is lower than that of LK and Farneback. As can be seen from the data in Table 2, the corresponding mean values of *MAE* and *APE* for LK are 29.13 and 7.94, while the mean values of *MAE* and *APE* for DFP-P-LK are 16.49 and 6.91, respectively, which are reduced by 43.39% and 12.97% with respect to those of LK. It indicates the effectiveness of the proposed feature point fusion detection and updating strategy in DFP-P-LK.

### 4.4. Experiments on DT-PLK-Based River Surface Flow Measurement

#### 4.4.1. Experiment on Dynamic Update Strategy of Feature Points

**Figure 6:**
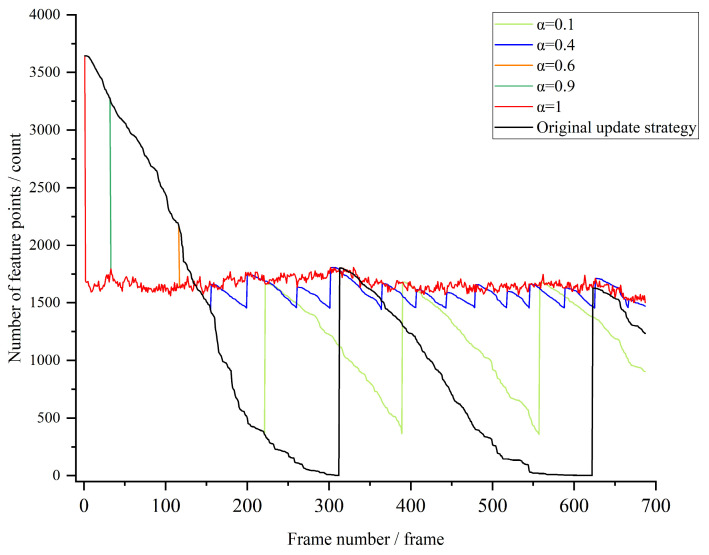
Original update strategy and changes in the number of tracked feature points under different α.

A water flow video is randomly selected as an example, which has 688 frames. We set the upper limit value $f_{max}$ of continuous processing video frames as 10 and use different update factors α to verify the feature point impact of continuous tracking, as shown in Figure 6. Figure 6 shows the feature point detection under the original update strategy and the dynamic update strategy with different update factors. The number of feature points tracked under the original update strategy shows a general trend of rapid decline, and when the number of successful tracking is 0, the feature point detection is repeated, and so on. The number of feature points is improved to different degrees after the dynamic update strategy is adopted.

**Figure 7:**
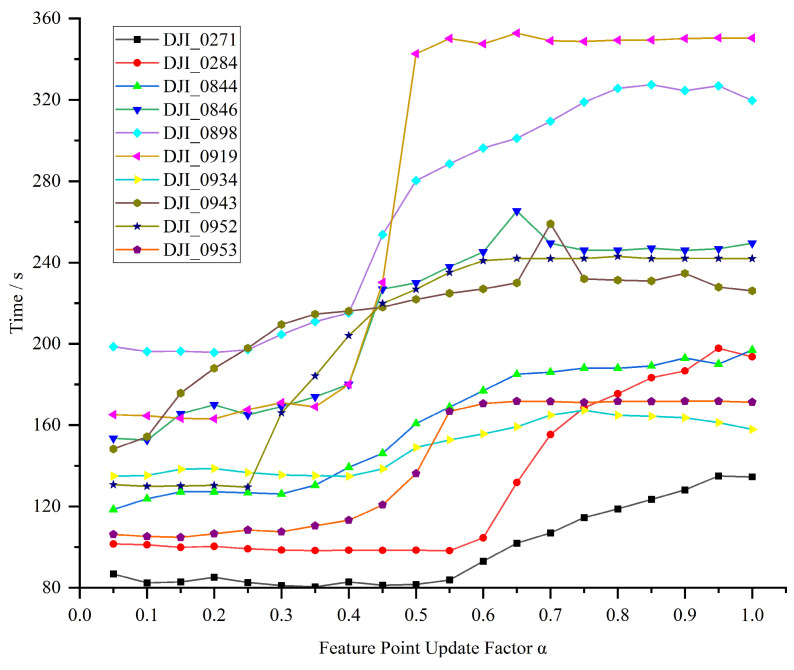
Relationship between runtime and update factor α for different videos.

In the case of unstable and volatile water surface texture features, the dynamic adjustment strategy can provide stable and reliable feature points to be tracked for subsequent optical flow matching and reduce the chance of false matching. However, in the case of improving the number of feature points, it is also necessary to take into account the time loss and choose a suitable update factor α. In order to find a suitable α, 10 water streaming videos were randomly selected for testing, and the relationship between their running time and update factor is shown in Figure 7. Due to the different lengths of different videos, only the trend of the processing time with the update factor α is dealt with here. Overall, the elapsed time increases with the increase of α, and the elapsed time in (0, 0.4) and (0.5, 1) increases slowly; the elapsed time in (0.4, 0.5) has an abrupt change trend. To summarize the number of feature points and the time consumed, α is set to 0.4.

#### 4.4.2. Water Flow Dataset Test and Result Analysis

**Figure 8:**
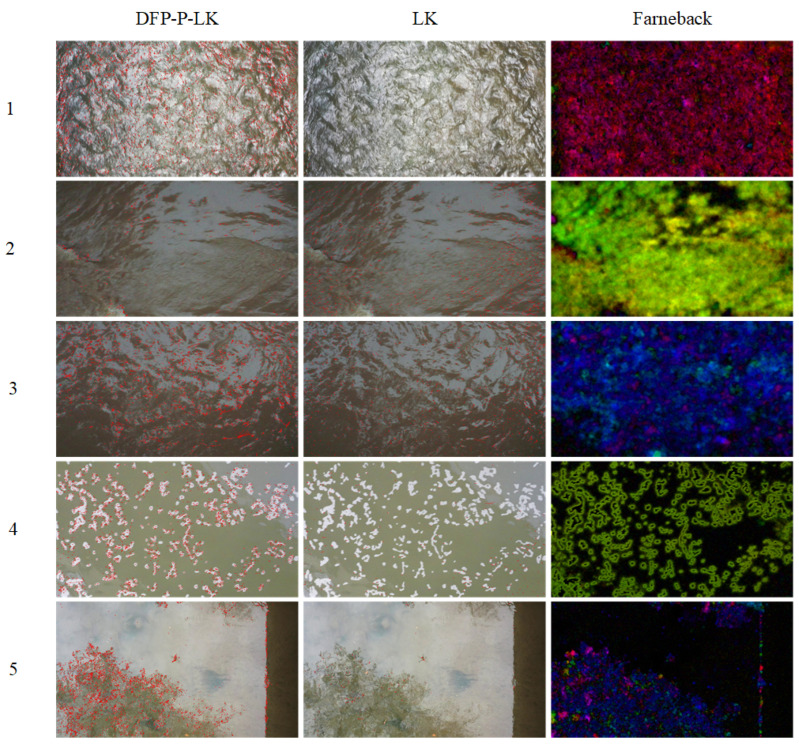
Example of optical flow visualization on a homemade water flow dataset.

In the test of the water flow video dataset, the accuracy of the detection speed was used as the index. Five of the representative videos were selected and analyzed. Numbers 1, 2, 3, 4, and 5 are, respectively, strong light and rapid water flow on a sunny day, weak light and turbid river water on a cloudy day, weak light and turbid river water after rain, smooth water flow with floating foam on a sunny day, and clear water flow with smooth flow and tree reflection on both sides of the riverbank on a sunny day. Table 3 shows the error analysis table of the river surface flow-velocity measurement, and the visualization of the optical flow under the algorithm is shown in Figure 8, where $v_{0}$ represents the average value of the results taken after several measurements by the water velocity meter, h represents the height of the lens from the surface of the water, v represents the results obtained from the image processing calculations in this experiment, and δ represents the relative error. The time interval between frames is 0.333 s.

The traditional LK optical flow method, Farneback method, and Template Matching method have poor detection accuracy and robustness in real water flow data, and the test results show large relative error fluctuations. In the non-clear and smooth water flow scenario, the relative errors of the LK optical flow method ranged from 13.71% to 34.62%, with an average relative error of 22.24%; the relative errors of the Farneback method fluctuated from 9.14% to 50%, with an average relative error of 21.48; and the relative errors of the template matching method ranged from 21.44% to 59.61%, with an average relative error of 34.885%. In contrast, the DFP-P-LK method performs much better in flow-velocity detection, with an average relative error of less than 15%, which is significantly superior to the DFP-P-LK method. The relative error was kept within 15%, which is significantly better than the traditional LK optical flow method, the Farneback method, and the template matching method.

### 4.5. Discussion

The experiments conducted on both the public dataset and the homemade water flow dataset indicate that the proposed DFP-P-LK method significantly outperforms the traditional LK and Farneback methods. However, these methods still exhibit some shortcomings in specific scenarios. In conditions where the water flow is clear and gentle, all three methods show relatively high errors. Specifically, the Farneback method has the lowest relative error at 47.06%, followed by the DFP-P-LK method at 64.71%, and the LK method at 94.12% is the highest. The sources of these errors can be attributed to several factors:

(1) Flowmeter measurement error. In low-speed flow conditions, the measurement error of the flowmeter is relatively high. This measurement error affects the evaluation of the algorithm’s performance, leading to higher relative errors;
(2) Difficulty in selecting feature points. In clear and gentle water flow conditions, it is challenging to find suitable and reliable feature points on the river surface for tracking and detection. This difficulty reduces the accuracy of detection and tracking, resulting in increased errors;
(3) Reflection interference. In scenarios with reflection interference from trees on both sides of the river, reflection interference becomes the main factor affecting the selection and matching accuracy of feature points. This interference makes feature point detection more difficult, thereby increasing errors.

To further enhance the performance of the DFP-P-LK method, the following improvement measures can be considered:

(1) Optimize feature point selection strategy. Enhance the feature point detection and tracking algorithms to improve robustness under varying lighting conditions and reflection interference. Additionally, in clear and gentle water flow conditions where finding suitable feature points is challenging, tools like drones can be used to scatter straw, bran, or leaves onto the water surface. These scattered materials create additional feature points that can be tracked and detected, thereby improving detection and tracking accuracy;
(2) Incorporate deep learning techniques. Combine deep learning models to automatically learn and extract more robust features, enhancing the accuracy and stability of optical flow estimation. Deep learning methods can better handle optical flow estimation problems in complex scenes and adapt to different environmental changes.

## 5. Conclusions

In this paper, the DFP-P-LK method is proposed by integrating the pyramid hierarchical structure, the dynamic feature point update strategy, and the feature point fusion detection mechanism, in order to improve the stability, accuracy, and robustness of the optical flow method in complex scenarios, so as to optimize the accuracy of the optical flow estimation. The experimental results show that the *MAE* of the DFP-P-LK method in FlyingChair is 16.49 and the *APE* is 6.91, which reduces the *MAE* and *APE* indexes by 43.39% and 12.97%, respectively, compared to the traditional LK algorithm. And the average error of constructing a flow measurement model based on DFP-P-LK is less than 15% on the homemade streamflow dataset. However, the performance of DFP-P-LK still needs to be further improved when dealing with complex situations such as light change and occlusion. The DFP-P-LK method will be further optimized in the future, including methods such as improving the feature point selection strategy and introducing deep learning techniques, to provide a more reliable and accurate solution to the motion estimation problem in complex scenes.

## Figures and Tables

**Table 1:** Information on homemade streamflow datasets.

River Section	Clear			Overcast			After Rain		Total
	Sunny	Turbid	Choppy	Sunny	Turbid	Choppy	Turbid	Choppy	
Zihu Creek	22	15	0	10	0	8	8	0	63
Sanhe River	5	0	4	0	6	0	0	0	19
Total	27	15	4	10	6	8	8	4	82

**Table 2:** *MAE* (in degrees) and *APE* (in pixels) under the Flying Chair dataset.

Serial Number	LK		DFP-P-LK		Farneback	
	*MAE*	*APE*	*MAE*	*APE*	*MAE*	*APE*
1	14.88	2.51	9.72	2.69	23.10	30.10
2	5.74	0.72	4.57	0.45	5.30	5.30
3	16.30	0.96	15.90	2.67	28.60	28.60
4	1.85	0.07	5.35	0.59	6.40	6.40
⋮	⋮	⋮	⋮	⋮	⋮	⋮
497	66.18	15.19	23.30	7.87	82.97	82.97
498	46.89	0.40	14.80	0.77	91.45	91.45
499	51.93	17.20	11.02	8.17	92.29	92.29
500	59.28	4.22	22.24	2.27	86.65	86.65
Average	29.13	7.94	16.49	6.91	30.61	31.23

**Table 3:** Error analysis table of river surface velocity measurement.

Serial Number	*v*_0_/(m∙s^−1^)	*h*/m	DFP-P-LK		LK		Farneback		Template Matching	
			*v*/(m∙s^−1^)	*δ*/%	*v*/(m∙s^−1^)	*δ*/%	*v*/(m∙s^−1^)	*δ*/%	*v*/(m∙s^−1^)	*δ*/%
1	1.75	6.37	1.61	8.00	1.51	13.71	1.59	9.14	1.38	21.14
2	0.90	7.80	0.82	8.89	0.73	18.89	1.00	11.11	0.59	34.44
3	1.15	6.23	0.98	14.78	0.90	21.74	0.97	15.65	0.87	24.35
4	0.52	6.70	0.61	17.31	0.70	34.62	0.78	50.00	0.83	59.61
5	0.17	5.68	0.28	64.71	0.32	94.12	0.25	47.06	0.05	70.58

## Data Availability

Data is unavailable due to privacy.
